# Short-term mechanical stretch fails to differentiate human adipose-derived stem cells into cardiovascular cell phenotypes

**DOI:** 10.1186/1475-925X-13-54

**Published:** 2014-05-01

**Authors:** Thais Girão-Silva, Vinicius Bassaneze, Luciene Cristina Gastalho Campos, Valerio Garrone Barauna, Luis Alberto Oliveira Dallan, Jose Eduardo Krieger, Ayumi Aurea Miyakawa

**Affiliations:** 1 Laboratory of Genetics and Molecular Cardiology, Heart Institute (InCor) - University of São Paulo School of Medicine, Avenue Dr. Eneas de Carvalho Aguiar, 44, São Paulo, SP 05403-000, Brazil

**Keywords:** Adipose-derived stem cell, Cell therapy, Stretch, Cell differentiation

## Abstract

**Background:**

We and others have previously demonstrated that adipose-derived stem cells (ASCs) transplantation improve cardiac dysfunction post-myocardium infarction (MI) under hemodynamic stress in rats. The beneficial effects appear to be associated with pleiotropic factors due to a complex interplay between the transplanted ASCs and the microenvironment in the absence of cell transdifferentiation. In the present work, we tested the hypothesis that mechanical stretch *per se* could change human ASCs (hASCs) into cardiovascular cell phenotypes that might influence post-MI outcomes.

**Methods:**

Human ASCs were obtained from patients undergoing liposuction procedures. These cells were stretched 12%, 1Hz up to 96 hours by using Flexercell 4000 system. Protein and gene expression were evaluated to identify cardiovascular cell markers. Culture medium was analyzed to determine cell releasing factors, and contraction potential was also evaluated.

**Results:**

Mechanical stretch, which is associated with extracellular signal-regulated kinase (ERK) phosphorylation, failed to induce the expression of cardiovascular cell markers in human ASCs, and mesenchymal cell surface markers (CD29; CD90) remained unchanged. hASCs and smooth muscle cells (SMCs) displayed comparable contraction ability. In addition, these cells demonstrated a profound ability to secrete an array of cytokines. These two properties of human ASCs were not influenced by mechanical stretch.

**Conclusions:**

Altogether, our findings demonstrate that hASCs secrete an array of cytokines and display contraction ability even in the absence of induction of cardiovascular cell markers or the loss of mesenchymal surface markers when exposed to mechanical stretch. These properties may contribute to beneficial post-MI cardiovascular outcomes and deserve to be further explored under the controlled influence of other microenvironment components associated with myocardial infarction, such as tissue hypoxia.

## Background

Myocardial infarction remains one of the major causes of morbidity and mortality worldwide. In cardiac ischemic injury, cardiomyocyte apoptosis, fibrous tissue deposition, and ventricular remodeling occur, which cause a decline in cardiac function [1-3]. In this context, cell therapy has been widely studied as a potential approach to improving cardiac repair and minimizing cardiac functional deterioration post-MI, but evidence for significant cell transdifferentiation is lacking, and it appears that other pleiotropic effects are occurring [4-7]. Adipose-derived stem cells (ASCs) are a promising and easily accessible choice for autologous cells to be used in cardiac repair [8-10]. ASCs have the potential to differentiate into multiple cell lineages including cardiovascular cells in vitro, even though significant transdifferentiation in vivo has not been demonstrated [11-15].

Data from our laboratory demonstrated that intramyocardial transplantation of ASCs in rats minimizes cardiac dysfunction under hemodynamic stress post-MI [16,17]. These effects are not observed when fibroblasts or myoblasts are injected alone but only when genetically modified to express Vascular Endothelial Growth Factor (VEGF) [18,19]. Indeed, we have recently demonstrated that ASCs produce VEGF [20], and we speculate that these beneficial influences on cardiac outcome may be attributed to the paracrine effects of released substances from ASCs under the influence of the microenvironment post-MI.

Stem cells implanted in the ischemic heart belong to a peculiar niche with specific chemical and physical cues caused by the hypoxic condition and the pulsatile contraction of this organ. In the present work, we tested whether mechanical force (stretch) *per se* can influence human adipose-derived stem cell properties that may lead to better post-MI cardiovascular outcomes.

## Methods

### Cell culture

#### Isolation, ex vivo expansion of hASCs

Human subcutaneous adipose tissues were obtained from patients undergoing liposuction procedures. All individuals gave informed consent to participate in the study, which was reviewed and approved by the University of São Paulo Ethics Committee (CAPPesq#:16688/06). Cells were isolated from adipose tissue as previously described [8]. In brief, harvested tissue was dissociated by digestion with collagenase IA and centrifuged. The pelleted cells were then recovered and plated onto 10-cm culture plates (NUNC, Rochester, NY). Plating and expansion medium consisted of Dulbecco’s modified Eagle medium (DMEM) low glucose with 10% Fetal Bovine Serum (FBS) and penicillin/streptomycin antibiotics (Invitrogen Corporation, Carlsbad, CA). After 24–72 hours, cultures were washed in Phosphate-Buffered Solution (PBS) to remove remaining erythrocytes and other unattached cells. Cells were maintained at 37°C with 5% CO_2_ in tissue culture dishes or flasks (Becton Dickinson, Franklin Lakes, New Jersey) until reaching 80% of confluence (usually within 5–7 days). Once 80% confluent, cells were detached with 0.5% trypsin-EDTA (Cultilab, São Paulo, SP, Brazil) and either re-plated at 1_×_10^4^cells/cm^2^ or used for experiments. This culture procedure is well characterized in our laboratory [20-22], and experiments were performed with cells up to 15th passage. Up to this passage, the cells display constant population doubling time and are non-senescent [22]. In addition, we have demonstrated adipogenic and osteogenic differentiation of these cells [20].

#### Cardiovascular cells

Endothelial cells (ECs) and SMCs were used as positive controls for some experiments. These cells were extracted from human Saphenous Vein segments (hSV) obtained from patients undergoing aortocoronary bypass surgery at the Heart Institute (InCor), University of São Paulo Medical School (Ethics Committee SDC 2454/04/074 – CAPPesq 638/04). ECs were isolated by incubation of hSV luminal surfaces with 1 mg/mL collagenase type II for 1 h at 37°C. The vessel was flushed with PBS and cell pellet was cultured in Human Endothelial – SFM with supplements (10% FBS, 20 ng/mL Fibroblast Growth Factor (FGF), 10 ng/mL Endothelial Growth Factor (EGF), 10 U/mL penicillin, 10 mg/mL streptomycin). Cells were characterized by positive immunofluorescence staining for von Willebrand Factor (vWF) (Sigma-Aldrich, St. Louis, MO), Vascular Endothelial Cadherin (VE-cadherin) (Cell Signaling Technology, Danvers, MA), and Cluster of Differentiation 31 (CD31) (Abcam, Cambridge, MA). SMCs were obtained according to an explant protocol. Briefly, the endothelial layer was removed by mechanical friction and small fragments of vessels were placed on six-well culture plates containing 3% gelatin. The fragments were cultured with DMEM high-glucose medium with 20% FBS and antibiotics (100 U/mL penicillin and 100 mg/mL streptomycin). SMCs derived from vessel fragments were isolated, expanded and characterized by hill-and-valley growth pattern and by immunofluorescence staining for Alpha Actin 2 (ACTA2) (Sigma-Aldrich).

### Stretch protocol

Primary cultures of hASCs were stretched by using the Flexercell 4000 cell stretching system (Flexcell International [23]). 1_×_10^5^ cells were plated in Bioflex plates covered with collagen type I and maintained in DMEM low glucose with 1% FBS (Hyclone) to stretch protocol (12% multiaxial stretch, 1 Hz, for 72 h and 96 h). Control non-stretched hASCs were also cultured on Bioflex plates with collagen I. During the experiment, the system was maintained at 37°C in humidified air with 5% CO_2_. At the end of the assay, the conditioned medium was frozen and the cells were washed with PBS and lysed for gene or protein expression analysis.

### Flow cytometry analysis

The immunophenotype of cultured hASCs was analyzed by flow cytometry using the flow cytometer FACSCalibur (Becton Dickinson, San Jose, CA). Cells were harvested and washed twice with PBS. Aliquots of 1_×_10^6^ cells were incubated for 15 minutes at room temperature with Fluorescein Isothiocyanate (FITC) or R-Phycoerythrin (PE)-conjugated monoclonal antibodies and washed twice in PBS containing 2% FBS and 0.1% sodium azide. Fluorochrome conjugated antibodies against CD29 and CD90 were used (BD Biosciences, San Jose, CA). Ten thousand events were acquired on a FACSCalibur flow cytometer, and Cell Quest software (BD Biosciences) was used for further analysis.

### Gene expression by RT–PCR and quantitative RT–PCR

Total RNA was isolated with Trizol Reagent (Invitrogen) and cDNA synthesis was performed with SuperScript III Reverse Transcriptase (Invitrogen) according to the manufacturer’s instructions. The amount of cDNA used for quantitative Reverse Transcription Polymerase Chain Reaction (qRT-PCR) (SYBR® Green PCR Master Mix-PE, Applied Biosystems, Life Technologies, Carlsbad, CA) is described on Table 1. The reaction was done in an ABI Prism 7700 Sequence Detection System (Applied Biosystems). All samples were assayed in triplicate. The control genes Glyceraldehyde-3-Phosphate Dehydrogenase (GAPDH) and Cyclophilin, which stay constant under experimental condition, were used to normalize the results. The Comparative Threshold (CT) cycle method was used for data analyses. CT indicates the fractional cycle number at which the amount of amplified target reaches a fixed threshold, and ΔCT is the difference in threshold cycle for the target gene (ACTA2, Transgelin (TAGLN), Myocyte Enhancer Factor 2C (MEF2C) and reference gene (GAPDH and Cyclophilin). The levels of the gene expression are expressed as 2^-ΔΔCT^; where ΔΔCT is the ΔCT value subtracted from ΔCT of static hASCs.

**Table 1:** Oligonucleotides primers used for qRT-PCR

**Target**	**Forward (5′-3′)**	**Reverse (5′-3′)**	**PCR product (bp)**	**cDNA (ng)**
ACTA2	TTCAATGTCCCAGCCATGTA	CATTGTGGGTGACACCATCT	109	15
Cyclophilin	ATGGTCAACCCCACCGTGT	TCTGCTGTCTTTGGGACCTTGTC	101	15
GAPDH	TGGTCTCCTCTGACTTCAACA	AGCCAAATTCGTTGTCATACC	118	25
MEF2C	ATCTGCCCTCAGTCAGTTGG	GGGTGGTGGTACGGTCTCTA	134	15
TAGLN	AACAGCCTGTACCCTGATGG	GCCCATCATTATTGGTCACT	212	25

Reverse Transcription Polymerase Chain Reaction (RT–PCR) was used to determine the gene expression in samples where real time RT-PCR did not reach acceptable efficiency of amplification: CD31, Kinase insert Domain Receptor (KDR), GATA binding protein 4 (GATA4), Calponin, Smooth Muscle Myosin Heavy Chain (SM-MHC), Myocardin (Myocd), Megakaryoblastic Leukemia (translocation) 1 (MKL1) and Myocardin-like Protein 2 (MKL2). The reaction was carried out using Taq polymerase under the following conditions: initial denaturation for 5 minutes at 95°C followed by cycles (24 – 35, Table 2) of denaturation for 15 seconds at 95°C, annealing for 1 minute at 60°C, extension for 1 minute at 72°C, and final extension for 10 minute at 72°C. The RT-PCR products were analyzed by electrophoresis with agarose gel. The bands were quantified by using ImageJ (http://rsb.info.nih.gov/ij/). GAPDH and Cyclophilin expression levels were used to normalize the results.

**Table 2:** Oligonucleotides primers used for RT-PCR, product size and cycles used

**Target**	**Forward**	**Reverse**	**PCR product (bp)**	**Cycles**
Calponin	CAGATGGGCACCAACAAAG	CATCTGCAGGCTGACATTGA	123	29
CD31	CCACTGCAGAGTACCAGCT	CACCTTGGATGGCCTCTTTC	80	35
Cyclophilin	ATGGTCAACCCCACCGTGT	TCTGCTGTCTTTGGGACCTTGTC	101	24
GAPDH	TGGTCTCCTCTGACTTCAACA	AGCCAAATTCGTTGTCATACC	118	24
GATA4	CTGTCATCTCACTACGGGCA	TAGCCTTGTGGGGAGAGCTT	122	34
KDR	TCAGAAGAGCTGAAAACTT	GAGCCTTCAGATGCCACAGA	80	34
MKL1	ACCGTGACCAATAAGAATGC	CATCTGCTGGCTTGAGGAAC	240	29
MKL2	ATTTCCAACGCTCACAGTCA	TTCACTGGCATTGTGGTGAT	146	29
Myocd	TTCCTGTGGATTCTGCTGTG	GGCTGTGAGGCTGAGTCATT	262	34
SM-MHC	CCATCCAGTTTCCTCTCCAC	GTCACTGAGTTGGCCCCTTCT	86	34

The primers were designed using the online software program Primer 3 (Primer 3, Ver.3, Whitehead Institute/MIT Center for Genome Research http://frodo.wi.mit.edu/). The amount of cDNA and cycles used in the reactions were defined as the linearity of the PCR amplification. All primers were analyzed by Primer-Blast (http://www.ncbi.nlm.nih.gov/tools/primer-blast/) to certify their specificity. The oligonucleotide primers used in this work for qRT-PCR and RT-PCR are in Tables 1 and 2, respectively.

ECs, SMCs and samples of necrotic human heart (approved by the University of São Paulo Ethics Committee – CAPPesq#: 0511/08) were used as positive controls.

### Western blot analyses

Cells were washed with PBS and lysed in lysis buffer (EDTA 1 mM, EGTA 1 mM, MgCl_2_ 2 mM, KCl 5 mM, HEPES 25 mM, PMSF 1 mM, DTT 2 mM, Triton X-100 0.1% and protease inhibitor cocktail (Sigma-Aldrich). After 10 minutes on ice, samples were centrifuged at 10,000 g for 10 minutes to remove cellular debris. Five to 40 μg of cell lysates were run on SDS-polyacrylamide gels and transferred. After electrophoresis, proteins were electro-transferred to PVDF membranes (Millipore, Billerica, MA) and transfer efficiency was monitored using 0.5% Ponceau S staining. The membrane was incubated in a blocking buffer (5% non-fat dry milk, 10 mM Tris–HCl, pH 7.6, 150 mM NaCl, and 0.1%Tween 20) for 2 h at room temperature and then probed with primary antibody. Each membrane was incubated overnight against a specific antibody: ACTA2 (Sigma- Aldrich), vWF (Sigma-Aldrich), Troponin I (HyTest,Turku, Finland), α-Sarcomeric Actin (Zymed, Life Technologies, Carlsbad, CA) and KDR (anti FLK1, # sc-504, Santa Cruz Biotechnology, Dallas, TX). After incubation with peroxidase conjugated secondary antibodies, detection was performed with enhanced chemiluminescence reagents (GE Healthcare). Protein levels of GAPDH (R&D) were used to normalize the results.

### Collagen gel lattice contraction assay

For measurement of contractility, cells were trypsinized from a monolayer culture and resuspended in DMEM low glucose at a density of 1_×_10^6^ cells/mL. The prepared cell suspension was added to collagen gel solution (BD Biosciences) to achieve a final concentration of 2.5 mg of collagen/mL and 4_×_10^5^ cells/mL as described elsewhere [24]. The mixture was poured into 12-well culture plates and incubated under standard culture conditions to polymerize the collagen cell lattices. After 1 hour, the lattices were mechanically released from the culture dishes by gently pipetting medium at the lattice-dish interface to initiate collagen gel contraction. The extent of gel contraction of each cell population was analyzed by measuring the dimensions of the lattice before release and after different time points up to 48 hours of release. The images were acquired by using a digital charge-coupled device camera, and the area of gel lattices was determined by using ImageJ (http://rsb.info.nih.gov/ij/). Relative lattice area was obtained by dividing the area at each time point by the initial area of the lattice. SMCs and ECs from hSV were used as positive and negative control, respectively.

### Nitric Oxide (NO) production

The NO production was evaluated by the amount of nitrite accumulation in the media of stretched and non-stretched cells using the colorimetric Griess assay as previously described [25]. Briefly, 100 μL of culture medium or nitrite standards (serial dilutions of NaNO_2_ in non-conditioned media; Sigma-Aldrich, St. Louis, MO) were mixed with 100 uL of Griess reagent, containing 50 μL of 1% sulphanilamide and 50 μL of 0.1% naphtylethelene-diamine-dihydrochloride (Sigma) in 2.5 M H_3_PO_4_. Absorbance was measured at 540 nm.

### Determination of cell-releasing factors

Cell culture medium from hASCs cultured in static or stretch condition for 96 hours were analyzed by RayBio® Human Angiogenesis Antibody Array C kit (able to detect 43 angiogenic factors), according to the manufacture’s manual. Briefly, array membranes were incubated with 2 mL of blocking buffer for 30 minutes at room temperature. Then, 4 mL of supernatant samples were placed into the membranes for 2 hours at room temperature. After 5 wash steps, 1 mL of biotin-conjugated antibodies were added to each array, and incubation was performed overnight at 4ºC. Membranes were washed and incubated with 1 mL of 1000-fold diluted HRP-conjugated streptavidin for 2 hours at room temperature. After washing, detection buffer was used for 2 minutes, and the array membrane was exposed to Hyperfilm (Amersham Bioscience) to detect the spots. The films were scanned and analyzed by densitometer using ImageJ (http://rsb.info.nih.gov/ij/). Positive controls present in each array were used to normalize all the soluble protein studied.

### Enzyme-Linked Immunosorbent Assay (ELISA)

VEGF, IL10, and IL8 secretion were detected by using ELISA. hASCs culture medium in a static or stretch condition (96 hours) were analyzed according to the manufacture’s instruction (R&D Systems). Briefly, each plate was incubated with 150 μL blocking buffer (PBS +2% Bovine Serum Albumin) for 2 hours at room temperature. After washing step, samples were incubated for 18 hours at 4°C. Another washing step was done prior to antibody enzyme conjugate, 2 hours at room temperature. Incubation with Streptavidin-HRP was done for reaction detection. Absorbance was read at 450 nm.

### Statistical analysis

Gene, protein expression and contraction assay are presented as mean ± standard error (SEM). Analysis of variance (two-way ANOVA, Bonferroni post-test) was used and p < 0.05 was considered significant for comparisons. All data are represented as fold induction relative to Static hASCs at 72 hours. NO production was also analyzed via two-way ANOVA with Tukey post-test (p < 0.05). Student *t* test was used for cytokine secretion evaluation and p < 0.01 was considered significant for comparisons.

## Results and discussion

**Figure 1:**
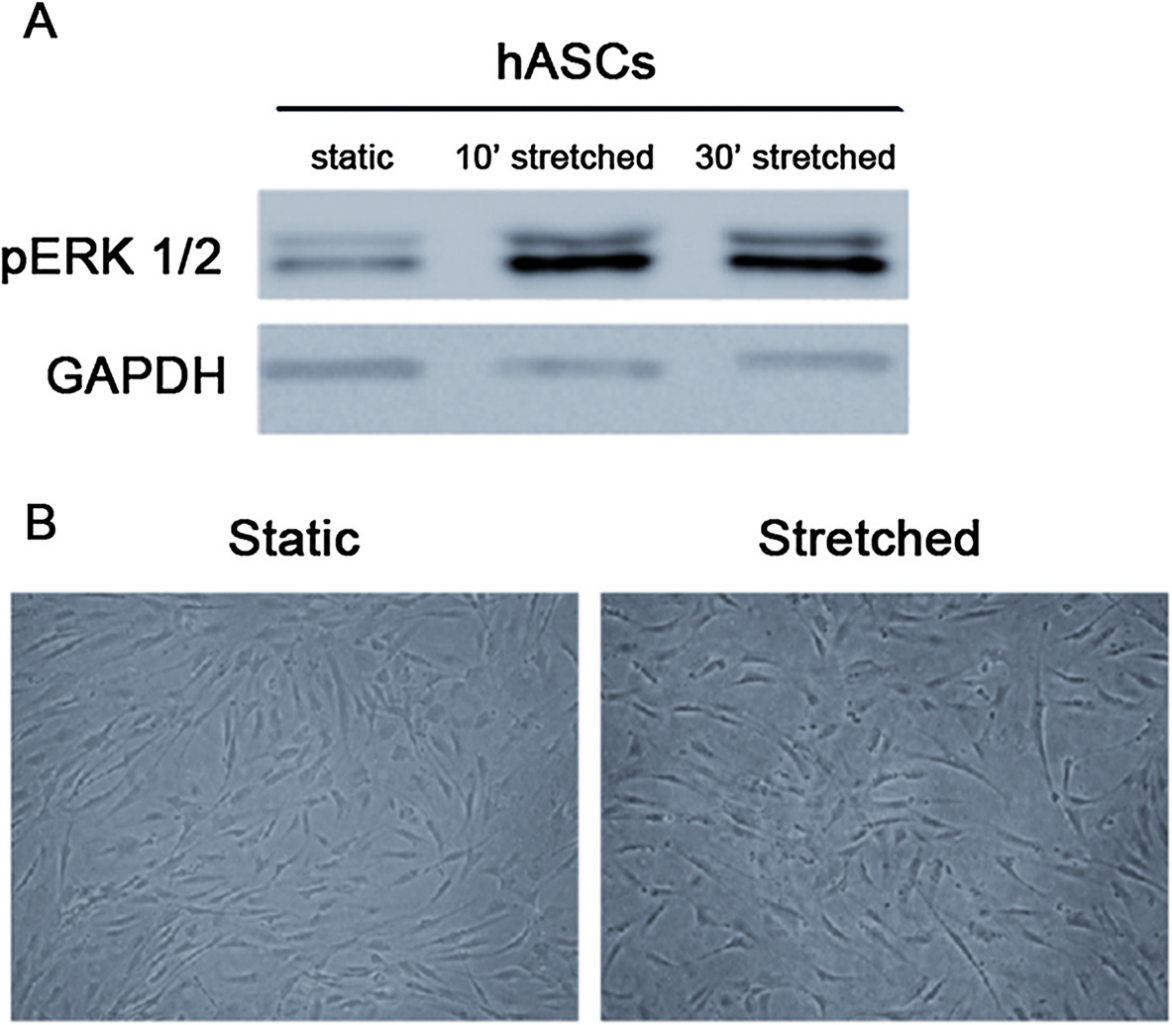
**Effects of mechanical stretch *per se *(12%, 1Hz) on hASCs morphology. (A)** Representative image of phosphorylated ERK expression after 10 and 30 minutes of cyclic strain, which demonstrates that stretching was efficiently applied to hASCs. **(B)** Mechanical stimulus up to 96 hours was not able to promote morphological changes, as demonstrated by microscopy analysis.

**Figure 2:**
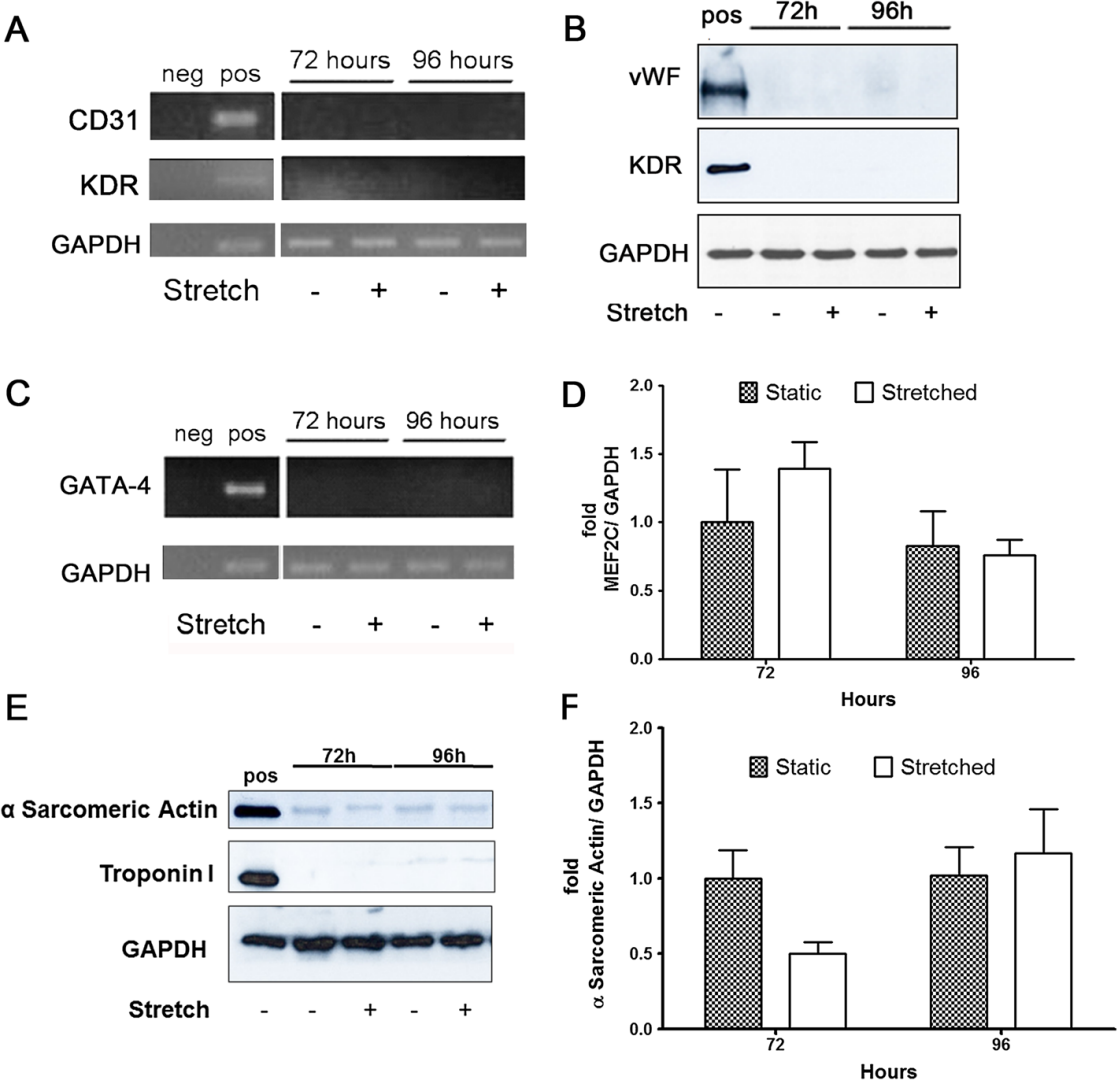
**Expression of Endothelial and Cardiomyocyte markers in hASCs that underwent stretching (12%, 1Hz). (A, B)** Endothelial markers in hASCs that underwent stretching for 72 and 96 hours. Saphenous vein endothelial cells. **(A)** Representative images of CD31 and KDR gene expression (n = 5). **(B)** vWF and KDR protein expression (n = 3). **(C-F)** Analysis of cardiomyocyte markers in hASCs stretched for 72 and 96 hours. **(C)** Representative image of GATA4 expression analyzed by RT-PCR (n = 3). **(D)** MEF2C gene expression was evaluated by qRT-PCR for 72 hours (n = 5) and for 96 hours (n = 6). Data are represented as mean ± SEM and relative to 72 hours static hASCs. **(E)** Troponin I (n = 3) and α-Sarcomeric Actin (n = 4) representative images of western blot experiment. **(F)** Quantification of α-Sarcomeric Actin protein expression (n = 4). Human heart myocardium was used as a positive control. Each bar represents means ± SEM.

**Figure 3:**
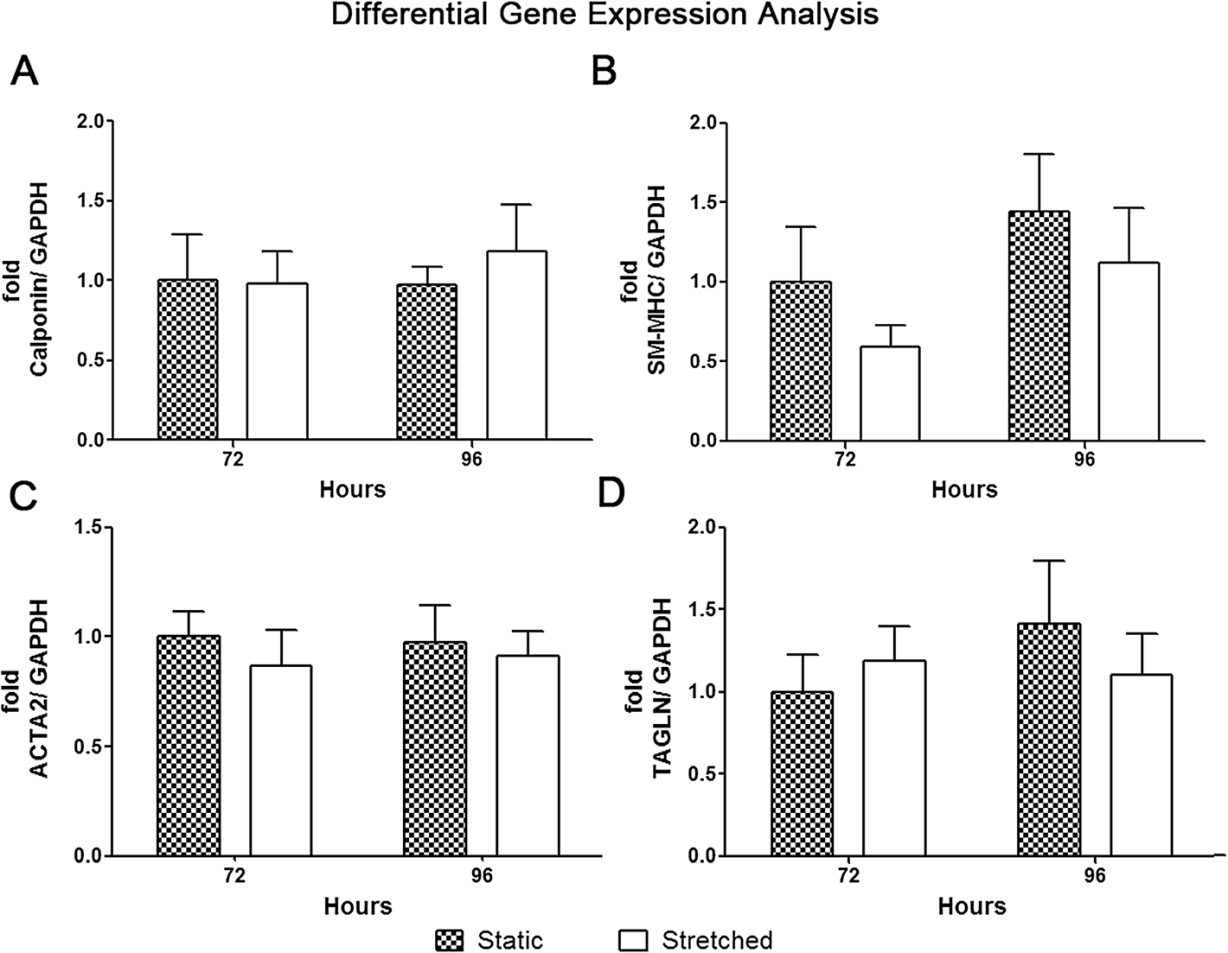
**Expression of Smooth Muscle Cell markers in hASCs that underwent stretching (12%, 1Hz). (A)** Calponin and **(B)** SM-MHC gene expression were analyzed by RT-PCR after strain for 72 (n = 5) or 96 hours (n = 6). Each bar represents means ± SEM. **(C)** ACTA2 and **(D)** TAGLN gene expression were evaluated by qRT-PCR after stretching for 72 (n = 5) or 96 hours (n = 6). Data are represented as means ± SEM. All data are represented as fold induction relative to Static hASCs at 72 hours.

**Figure 4:**
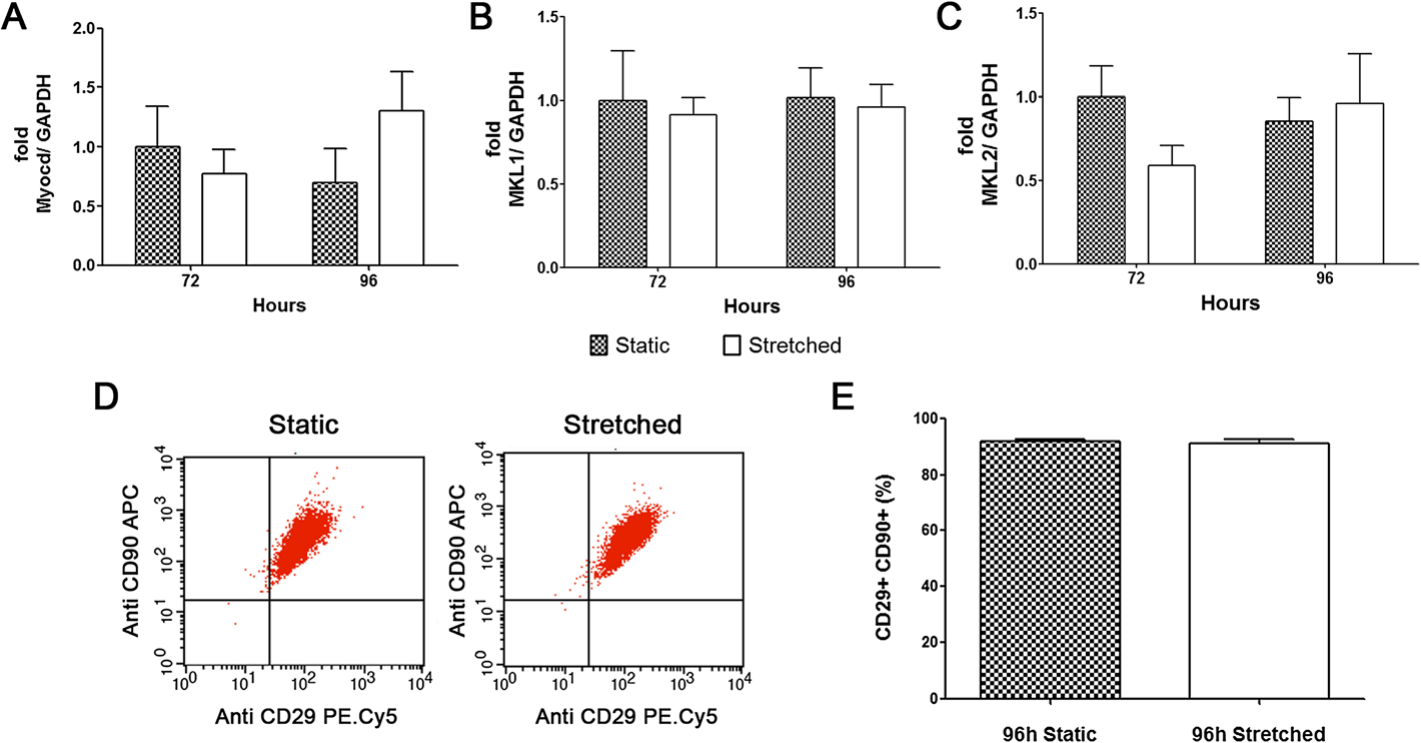
**Expression of myogenic co-factors and mesenchymal markers in stretched hASCs (12%, 1Hz). (A)** Myocd, **(B)** MKL1 and **(C)** MKL2 gene expression were analyzed by RT-PCR after strain for 72 (n = 5) or 96 hours (n = 6). Each bar represents means ± SEM. **(D)** Representative dot blot and **(E)** percentage quantification of CD29/CD90 positive mesenchymal markers in static and stretched hASCs for 96 hours analyzed by flow cytometer (n = 4). Bar represents means ± SEM.

Mechanical stretch up to 96 hours was efficiently applied to human adipose-derived stem cells, indicated by increased expression of phosphorylated ERK (Figure 1A) as previously reported [26]. After stimulation, these cells were analyzed by contrast phase microscopy. No morphological change was observed, with the characteristic fibroblast-like phenotype maintained (Figure 1B). Differentiation into cardiovascular cell phenotypes was assayed by gene and protein expression of respective molecular markers (Figures 2, 3 and 4A-C). Endothelial markers (CD31, KDR, and vWF), analyzed by gene and protein expression, were not detected in static or in stretched cells (Figure 2A and 2B). Similarly, no induction of cardiomyocyte markers occurred as a result of stretching (GATA-4, MEF2C, troponin I, α Sarcomeric Actin – Figure 2C-F). Although mechanical stretch did not change these markers, it is interesting to notice that hASCs naturally demonstrated MEF2C gene expression (Figure 2D) and α Sarcomeric Actin protein expression (Figure 2E, quantification in Figure 2F). Our data also indicate basal expression of SMC markers (ACTA2, TAGLN, SM-MHC, Calponin) and myogenic co-factor markers (Myocd, MKL1, MKL2) in static hASCs and this expression pattern was not modified by mechanical stimulation (Figures 3 and 4A-C). The expression of CD29/CD90, two immunophenotypic mesenchymal stem cell markers, is maintained in hASCs after mechanical stimulation, as shown by flow cytometer (Figure 4D). In fact, there was no difference in CD29/CD90 expression in stretched cells compared to static hASCs (Figure 4E).

It has been shown that ASCs have the potential to differentiate into ECs [11,27] SMCs [24,28,29] and cardiomyocytes [12-15]. FGF, VEGF and contact with methylcellulose matrix have all been reported to induce ECs phenotype [11]. Moreover, ASCs also display the potential to differentiate towards the smooth muscle cell line, once SMC markers are expressed after chemical treatments, such as angiotensin II and Sphingosylphosphorylcholine (SPC) [24,28]. Additionally, ASCs have already been demonstrated to differentiate *in vitro* into cardiomyocytes [12-15]. Cardiac markers were spontaneously expressed in hASCs after being in culture for 20 days via the paracrine effect of VEGF secretion [15].

Evidence indicates that mechanical stretch can induce differentiation of ASCs into the cardiovascular cell phenotype. Balata *et al.*[30] showed that stretch alone could induce myogenic differentiation in rat ASCs. The authors demonstrated that cyclic uniaxial strain (10%, 1Hz) for 24 hours increased the gene expression of myogenic markers (Myod, Myog and Myh2), and this pattern was even greater when associated with chemical factors. Recently, similar results have also been reported in hASCs. Protein expression (by immunofluorescence) and the presence of multi-nucleated myotubes showed myogenic differentiation after 7 days (up to 21) of uniaxial strain (1 hour/day, 11%, 0.5Hz) [31]. In addition, Lee *et al.* showed a reduction in protein expression of some smooth muscle markers (ACTA2 and Calponin) in hASCs after 7 days [32]. The same mechanical stimulation was performed (cyclic uniaxial strain); however, the strain was constant at 10%, 1Hz.

The potential differentiation of ASCs into cardiomyocytes by mechanical stimulation has also been demonstrated. Rat bone marrow derived stem cells that underwent cyclic uniaxial strain for 24 hours (up to 72 h) were differentiated into cardiomyocytes demonstrated by an increase in gene expression (GATA4, MEF2C, β-MHC, NKx2.5) [33].

Thus, although there is evidence of ASCs differentiation into cardiovascular cells in vitro, our data indicate that stretch *per se* under the described experimental conditions is not sufficient to induce any of these reported phenotypes. The culture of hASCs has been extensively characterized in our laboratory. We have demonstrated that human ASCs display constant population doubling time, are non-senescent up to the 15th passage and have the potential to differentiate into adipogenic and osteogenic lineages [22]. In the present work, most of the experiments were performed using cells between passage 4 and 8. No difference was observed in the results obtained from cells at different passages (data not shown). Corroborating our results, Yong Guo *et al.*[34] demonstrated that mechanical stretch (8% elongation for 10 days) has no effect on rat ASCs’ differentiation into cardiomyocytes, even though this stimulus accelerated the differentiation of rat ASCs treated with 5-azacytidine, highlighting the complex interplay between a variety of factors associated with the differentiation.

**Figure 5:**
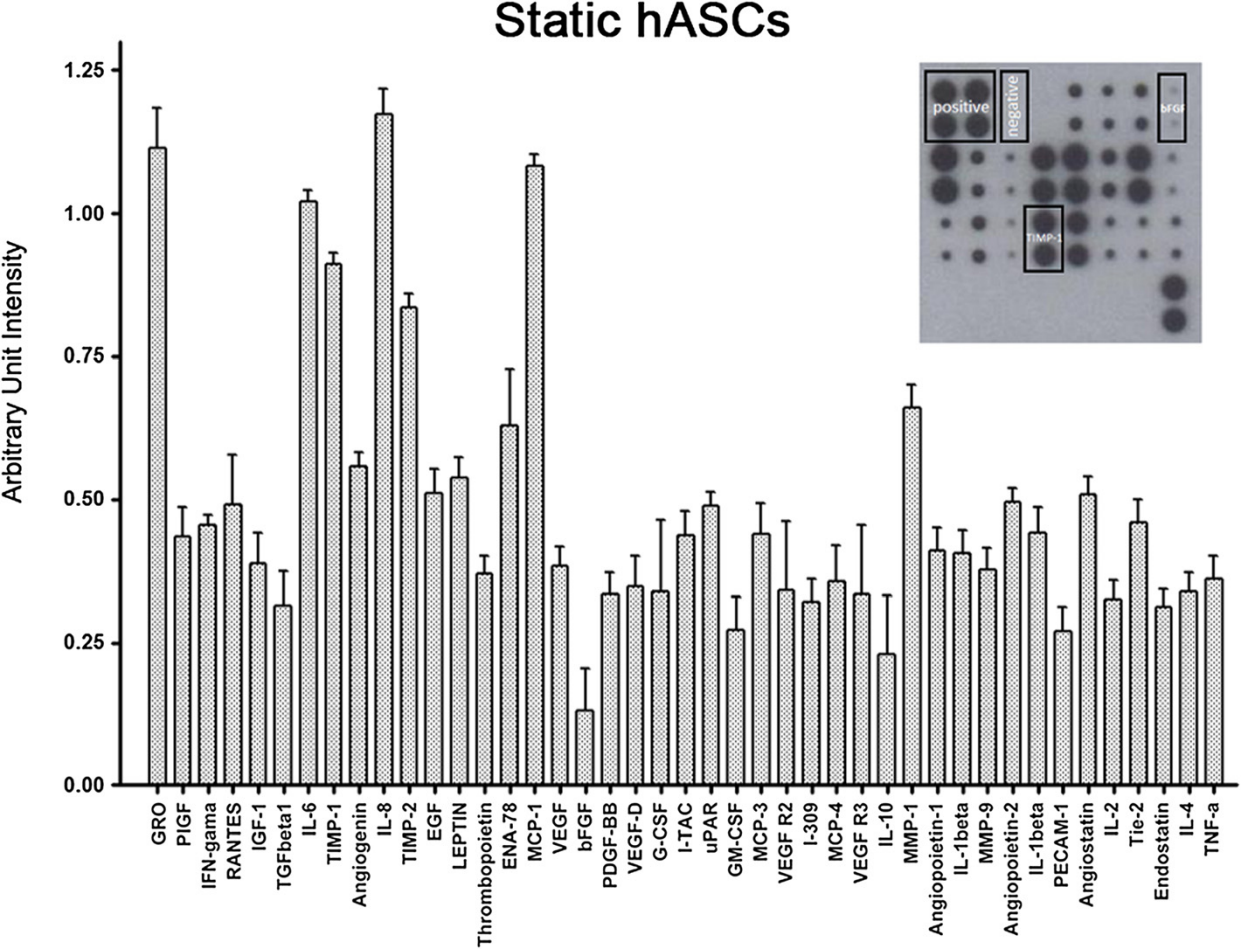
**Basal cytokine profile secreted by hASCs.** The upper right insert is a representative image of the array membrane incubated with conditioned medium of hASCs under static condition. All the 43 angiogenic factors were plotted as arbitrary unit intensity normalized by positive controls present in each array. Each bar represents mean ± SEM of 4 independent experiments.

**Figure 6:**
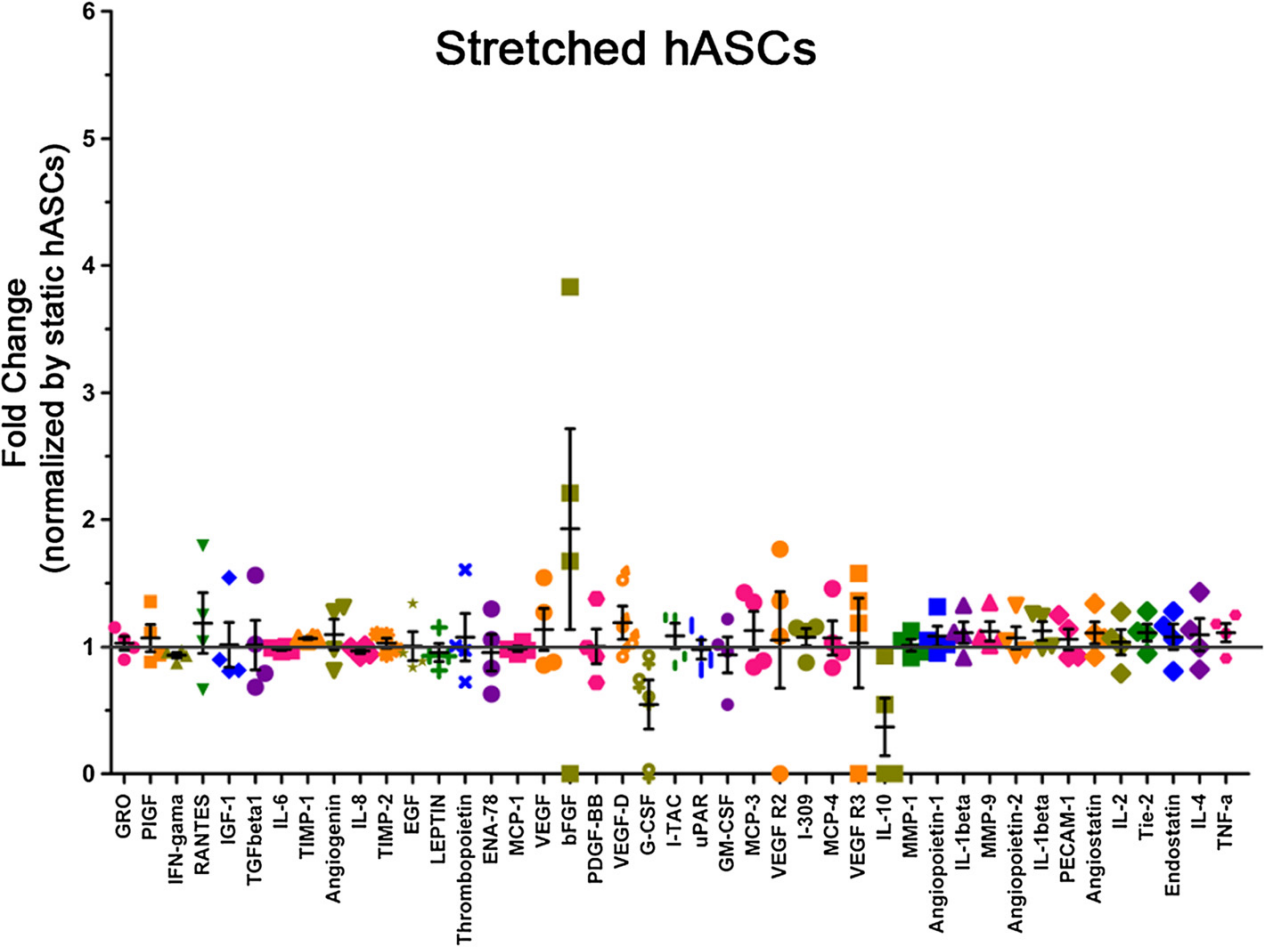
**Cytokine profile secreted by hASCs that underwent stretching (12%, 1Hz) for 96 hours.** Data are represented as fold change secretion of stretched hASCs compared to static hASCs (n = 4, independent experiments). The gray line represents the level of secretion of static hASCs (Figure 5).

We have previously demonstrated that shear stress *per se* is also not able to induce endothelial markers in human and porcine ASCs, but it induced NO-dependent VEGF production [20] [Dariolli R, data unpublished]. In contrast, the present results demonstrate that NO release was not significantly different when comparing static and stretched cells for 72 and 96 hours (static 72 h: 0.51 ± 0.10; stretched 72 h: 0.49 ± 0.15; static 96 h: 0.55 ± 0.03; stretched 96 h: 0.80 ± 0.07 nmol/10^4^ cells; n = 8 independent experiments). To assess secreted factors that potentially can influence processes triggered by tissue ischemia, we verified the profile of 43 cytokines secreted by hASCs while stretched. Static hASCs showed an exuberant basal cytokine secretion profile including GRO, IL-6, IL-8, Tissue Inhibitor of Metalloproteinase 1 (TIMP-1), TIMP-2 and Monocyte Chemotactic Protein 1 (MCP-1). On the other hand, Transforming Growth Factor Beta 1(TGFβ1), bFGF, Granulocyte Macrophage Colony Stimulating Factor (GM-CSF) and IL-10 displayed lower expression (Figure 5). Interestingly, there was no alteration in the cytokine expression profile of hASCs exposed to 96 hours of mechanical stretch (Figure 6). The ELISA assay was also used to detect VEGF, IL8 and IL10, and no difference was observed in hASCs both static and stretched for 96 hours, confirming data obtained with antibody array (Table 3).

**Table 3:** VEGF, IL8 and IL10 secretion by hASCs (96 hours) detected by ELISA

**Cytokine**	**Static hASC (pg/mL)**	**Stretched hASC (pg/mL)**	**n**
VEGF	110.9 ± 68.8	132.8 ± 55.7	8
IL8	2513.2 ± 139.3	2523.5 ± 284.1	8
IL10	14.56 ± 2.29	11.32 ± 5.39	8

Similar to that described for mesenchymal stem cells from blood cord and bone marrow [35,36], hASCs display an exuberant secretion capacity (Figures 5 and 6). This is consistent with the finding that ASCs from different sources of adipose tissue secrete soluble angiogenic and anti-apoptotic factors able to improve vascular ischemic diseases [37,38]. In addition, it has been reported that expression of pro-inflammatory cytokines after myocardial infarction can be beneficial to cardiac repair and the balance of those cytokines is important in initiating the angiogenic process [39-41]. Angiogenesis begins after low oxygen tension and requires enzymatic degradation of extracellular matrix, endothelial cell proliferation and migration to form new vessels [42-45]. In this context, the balance between MMP (Matrix Metalloproteinase) and TIMP activity will determine the ongoing angiogenic process. Moreover, TIMP have been considered multifunctional proteins, which besides their metalloproteinase inhibitor activity have the ability to induce growth of endothelial cells and anti-apoptotic activity [46-50]. Since hASCs showed a great amount of TIMP-1 and TIMP-2 (Figure 5) release, one can speculate that these cells might also contribute to the angiogenesis observed during tissue remodeling.

**Figure 7:**
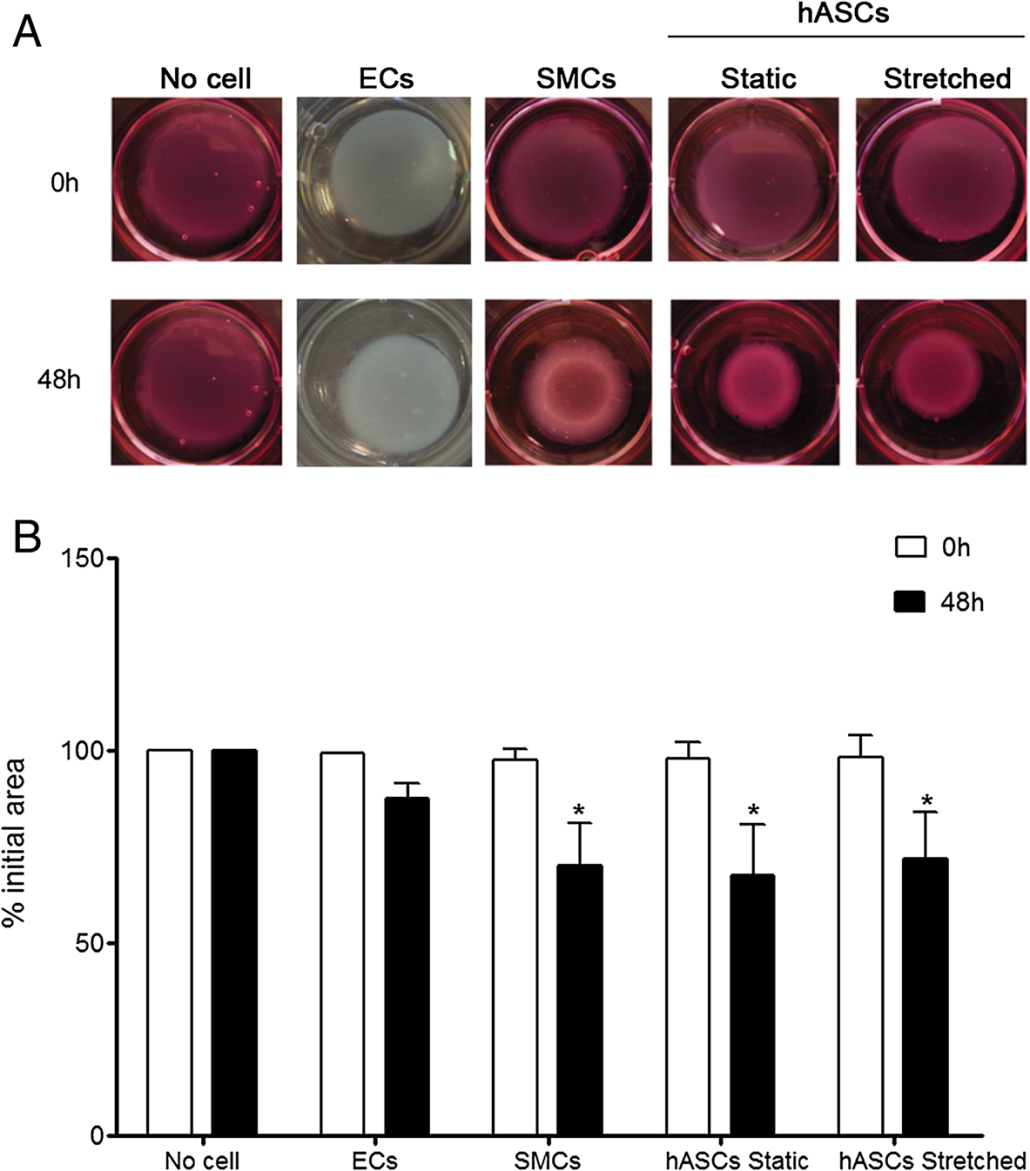
**Contraction assay of hASCs that underwent mechanical stimulation (12%, 1Hz). (A)** Illustrative image from collagen gel discs with no cells and with endothelial cells (ECs), smooth muscle cells (SMCs), static hASCs or 96 hours stretched hASCs. ECs and SMCs from human saphenous vein were used as negative and positive controls of contraction, respectively. **(B)** Quantification of gel disc contraction was analyzed after 48 hours (hASCs, n = 7; SMCs n = 4; ECs, n = 2). Relative lattice area was obtained by measuring the final dimensions in comparison with its initial area. The bars represent means ± SD and * indicates p < 0.01 compared to 0 h. Sample sizes indicate data from independent experiments.

hASCs that underwent mechanical stimulation for 96 hours were tested by means of functional contraction assay in collagen gel (Figure 7A). We observed that these cells have contraction ability, corroborating data described by other laboratories [24,28,29,51]. Indeed, this is consistent with the gene expression profile of some contractile proteins studied in this work (Figures 3, 4A-C), and justifies the basal contraction potential similar to that of SMCs (32.3 ± 13% vs. 29.9 ± 10.9%, at 48 hours, n = 4). Mechanical stretch for 96 hours resulted in no change in this phenotype (28.1 ± 12.2%) (Figure 7B). On the other hand, it has been reported that hASCs chemical treatment, such as SPC or TGFβ, promotes increments in cell contraction [28,51]. Kim *et al.* demonstrated that U46619 (Thromboxane A2 mimetic) treatment for 96 hours induces hASCs expression of some contractile proteins, which was associated with an almost 60% increase in contractility of these cells [29]. The native contractile properties of hASCs could limit infarct growth and prevent cardiac function deterioration. These findings could explain, at least in part, the beneficial cardiac post-MI outcomes associated with ASCs transplantation using different experimental models [52-54].

It is widely accepted that ASCs display secretion capacity and contraction properties as we also demonstrate in the present work. In addition to that, we demonstrate that mechanical stretch *per se* was not able to modify their properties. Since implanted cells in the ischemic heart are in a peculiar niche caused by the hypoxic condition and the pulsatile contraction of this organ, we are currently investigating the secretory profile of these cells when stimulated with mechanical stretch and hypoxia, as seen under the tissue ischemic microenvironment.

## Conclusions

Taken together, we provide evidence that hASCs secrete a variety of factors and display contractile properties. These phenotypic characteristics may contribute to the beneficial pleiotropic effects on cardiac post-MI outcomes associated with ASCs transplantation. Even though stretch *per se* did not influence these properties, the association of mechanical and chemical stimuli, as seen under the tissue ischemic microenvironment, deserves to be further explored to better understand the beneficial pleiotropic effects on cardiac post-MI outcomes associated with ASCs transplantation.

## Abbreviations

ACTA2: Alpha actin 2; ASCs: Adipose derived-stem cells; CD31: Cluster of differentiation 31; CT: Comparative threshold; DMEM: Dulbecco’s modified eagle medium; ECs: Endothelial cells; FBS: Fetal bovine serum; FGF: Fibroblast growth factor; GAPDH: Glyceraldehyde-3-phosphate dehydrogenase; GATA4: GATA binding protein 4; hASCs: Human adipose derived-stem cells; hSV: Human saphenous vein segments; KDR: Kinase insert domain receptor; MEF2C: Myocyte enhancer factor 2C; MI: Myocardium infarction; MKL1: Megakaryoblastic leukemia (translocation) 1; MKL2: Myocardin-like protein 2; Myocd: Myocardin; NO: Nitric oxide; PBS: Phosphate-buffered solution; qRT-PCR: Quantitative reverse transcription polymerase chain reaction; RT-PCR: Reverse transcription polymerase chain reaction; SMCs: Smooth muscle cells; SM-MHC: Smooth muscle myosin heavy chain; SPC: Sphingosylphosphorylcholine; TAGLN: Transgelin; TGFβ: Transforming growth factor beta; TIMP: Tissue inhibitor of metalloproteinase; VE-cadherin: Vascular endothelial cadherin; VEGF: Vascular endothelial growth factor; vWF: Von willebrand factor.

## Competing interests

The authors declare no financial or any other conflict of interest involved in this work.

## Authors’ contributions

TG-S did the hASCs culture, stretch experiments, processed the samples for gene and protein expression, flow cytometry analysis, NO production measurement, participated in RT-PCR, qRT-PCR, carried out the collagen gel contraction assay, human antibody array detection, ELISA, participated in the statistical analysis, design of experiments and development of the manuscript. VB participated in the hASCs isolation and culture, flow cytometry analysis, NO production measurement, ELISA, statistical analysis and design of experiments. LCGC participated in the gene expression experiments. VGB carried out the western blot experiments and participated in the design of the study. LAOD participated in the design of experiments and revision of the manuscript. AAM and JK conceived the study, participated in the design of experiments, coordinated on and revised the manuscript, and helped with funding acquisition. All authors read and approved the final manuscript.
